# 2'-Hydroxyflavanone activity *in vitro* and *in vivo* against wild-type and antimony-resistant *Leishmania amazonensis*

**DOI:** 10.1371/journal.pntd.0006930

**Published:** 2018-12-06

**Authors:** Luiza F. O. Gervazoni, Gabriella Gonçalves-Ozório, Elmo E. Almeida-Amaral

**Affiliations:** Laboratório de Bioquímica de Tripanosomatídeos, Instituto Oswaldo Cruz, Fundação Oswaldo Cruz, Manguinhos, Rio de Janeiro, Brazil; Pasteur Institute of Iran, ISLAMIC REPUBLIC OF IRAN

## Abstract

**Background:**

To overcome the current problems in leishmaniasis chemotherapy, natural products have become an interesting alternative over the past few decades. Flavonoids have been studied as promising family of compounds for leishmaniasis treatment. 2’-Hydroxyflavanone (2HF) is a flavanone, a class of flavonoid that has shown promising results in cancer studies. In this study, we demonstrated the effects of 2HF *in vitro* and *in vivo* against wild-type and antimony-resistant *Leishmania amazonensis* promastigotes.

**Methodology/Principal findings:**

2HF was effective against promastigotes and the intracellular amastigote form, decreasing the infection index in macrophages infected with wild-type and antimony-resistant promastigotes, but it was not toxic to macrophages. *In silico* analysis indicated 2HF as a good oral candidate for leishmaniasis treatment. *In vivo*, 2HF was able to reduce the lesion size and parasite load in a murine model of cutaneous leishmaniasis using wild-type and antimony-resistant promastigotes, demonstrating no cross-resistance with antimonials.

**Conclusions/Significance:**

Taken together, these results suggest 2HF as a potential candidate for leishmaniasis chemotherapy for cutaneous leishmaniasis caused by both wild-type and antimony-resistant *Leishmania* species by oral administration. Furthermore, studies should be conducted to determine the ideal dose and therapeutic regimen.

## Author summary

Leishmaniasis is a parasitic disease endemic to 98 countries, affecting more than 12 million people globally, and there are more than 350 million people in risk areas. Although there are many drugs available as alternatives for leishmaniasis treatment, they remain mostly ineffective, expensive and longstanding, in addition to generating side effects and resistance. Antimonial resistance is currently one of the biggest obstacles in leishmaniasis chemotherapy. Due to the poor chemotherapy scenario and the need for a drug able to overcome resistance problems and therapeutic failures, natural products have become an important alternative for leishmaniasis treatment. Here, we evaluated the antileishmanicidal activity of 2HF *in vitro* and *in vivo* against wild-type and antimony-resistant *L*. *amazonensis* cells. 2HF inhibited the cellular proliferation of promastigotes and the intracellular amastigote form in a dose-dependent manner in both wild-type and antimony-resistant cells. Furthermore, 2HF reduced the lesion size and parasitic load in a murine model of cutaneous leishmaniasis using wild-type and antimony-resistant promastigotes without altering hematological parameters and serological toxicology markers. This is the first time that the activity of a flavonoid on the antimony-resistant *L*. *amazonensis* has been demonstrated *in vitro* and *in vivo* by the oral route.

## Introduction

Known as neglected tropical disease globally, leishmaniasis is endemic to 98 countries, and there are more than 350 million people in risk areas. It deserves attention due to the wide variety of clinical manifestations and its high annual incidence [1]. This disease, which is caused by over 20 species of pathogenic parasites of the genus *Leishmania*, is divided into two major clinical manifestations: the visceral form (VL), which causes death by affecting internal organs such as the spleen and liver, and the cutaneous form (CL), which is subdivided into many forms that affect the skin and mucous membranes [2]. Even though it does not lead to death, the cutaneous form causes many social problems for patients. Among all the species that cause CL, *Leishmania amazonensis* is known to induce a wide spectrum of clinical manifestations, including the most aggressive mucosal form [3].

Leishmaniasis treatment has been mostly based on pentavalent antimonials as the first choice for over 70 years. Amphotericin B is the second choice, but in cases of therapeutic failure, it becomes the first treatment choice [4]. Miltefosine, the first oral drug for leishmaniasis, has become an important alternative; however, its use is not licensed all over the world. Although there are many drugs available as alternatives for leishmaniasis treatment, they remain mostly ineffective, expensive and longstanding, in addition to generating side effects and resistance [5].

Antimonial resistance is currently one of the biggest obstacles in leishmaniasis chemotherapy. It has been described since antimonials began to be used in clinic, and it is one of the major causes of therapeutic failure [6,7]. Over the decades, antimonial resistance became an emerging worldwide problem, embracing visceral and cutaneous leishmaniasis, being reported not only in India and South Africa, as the first cases, but in African continent recently [8]. These reports combined with antimonial extensive use as first line treatment in several countries yet, suggest a resistance progression leading a warning to the world. The mechanism of resistance has been exhaustively studied and is strongly associated with the overexpression of ABC-family drug transporters and MDR genes, indicating the possibility of cross-resistance [9,10]. Other reference drugs have also demonstrated resistance generation, such as miltefosine [11,12] and pentamidine [13].

With the current lack of a vaccine, the poor chemotherapy scenario and the need for a drug able to overcome resistance problems and therapeutic failures, natural products, mostly plant secondary metabolites, have become important alternatives for leishmaniasis over the past few decades [14–16]. Flavonoids are a group of secondary metabolites present in fruits, vegetables, wine and coffee and are classified into flavones, flavanones, flavonoids, flavonols, anthocyanins, isoflavonoids and chalcones [17].

**Fig 1:**
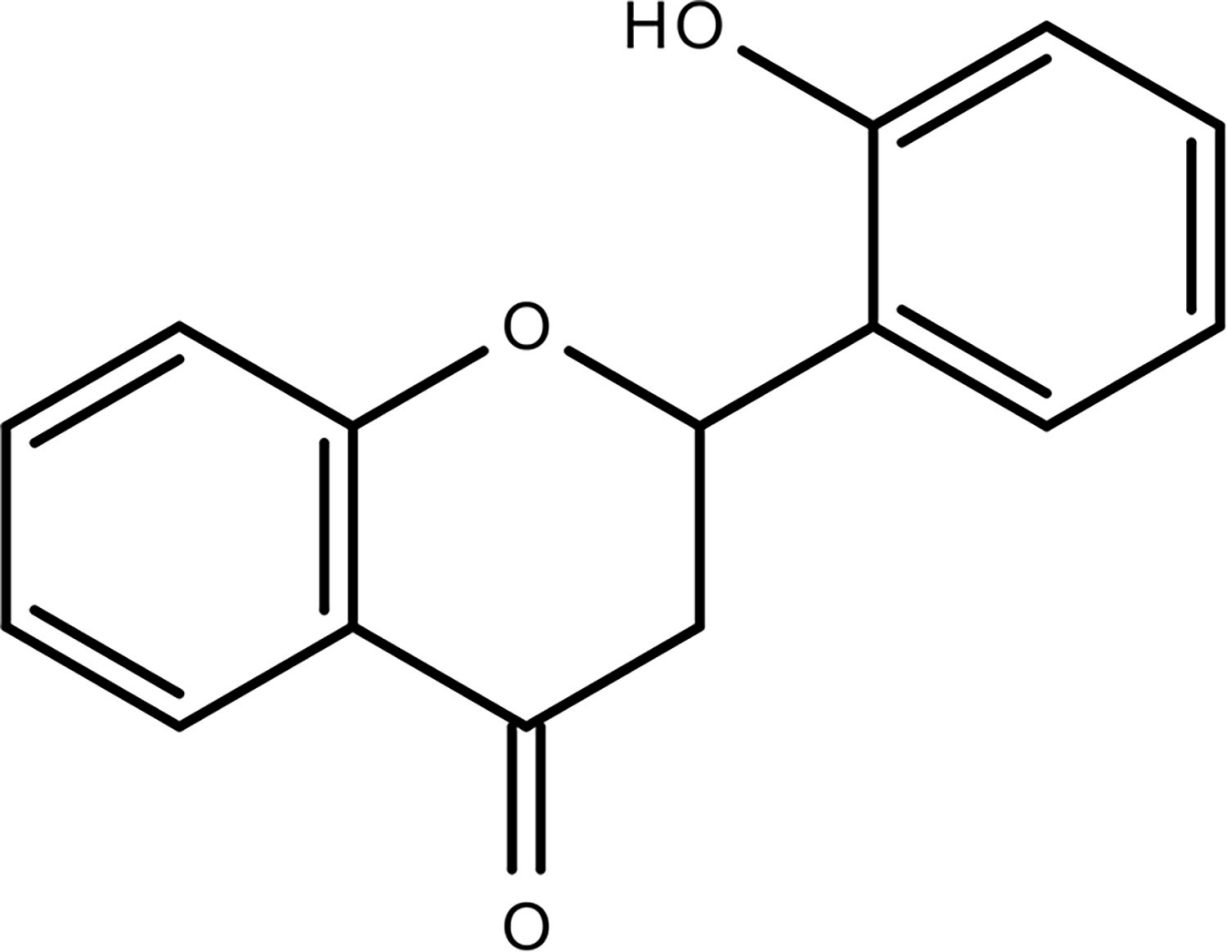
Chemical structure of 2HF used in this study.

Activity of different flavonoids against *Leishmania* has been demonstrated. Quercetin, apigenin and epigallocatechin *O*-3 gallate have been reported as promising oral candidates to leishmaniasis chemotherapy in different species of cutaneous leishmaniasis[18–23]. 2’-Hydroxyflavanone (2HF, Fig 1) is a flavanone, a class of flavonoids present in fruits, especially in citric fruits such as oranges. It has been studied as a possible alternative for many types of cancer treatment, such as renal, colon, and lung cancer and osteosarcomas. In cancer cells, the mechanism of action of 2HF remains unknown, appearing to follow different pathways according to cell type. It was able to induce apoptosis, inhibit the differentiation of tumor markers and prevent the vascularization, proliferation and migration of cancer cells [24–26]. In the present study, we evaluated the leishmanicidal activity of 2HF *in vitro* and *in vivo* against wild-type and antimony-resistant *L*. *amazonensis* cells.

## Materials and methods

### Compounds

2HF (≥98% purity; lot SLBT8413), Schneider's Drosophila medium, RPMI-1640 medium, potassium antimony (III) tartrate hydrate, penicillin and streptomycin were obtained from Sigma-Aldrich (St. Louis, MO, USA). Fetal calf serum was obtained from Cultilab (Campinas, SP, Brazil). All other reagents were purchased from Merck (São Paulo, Brazil). Deionized distilled water was obtained using a Milli-Q system (Millipore Corp., Bedford, MA, USA) and was used to prepare all solutions. Endotoxin-free sterile disposable supplies were used in all experiments. 2HF was prepared in dimethyl sulfoxide (DMSO) and diluted in culture medium such that the solvent concentration did not exceed 0.2% (v/v) in the final solution. In the control samples (absence of 2HF), a similar volume of vehicle (DMSO 0.2% v/v) was added to the cells. Meglumine antimoniate (Glucantime, Sanofi, São Paulo, Brazil) was provided by Evandro Chagas National Infectology Institute, FIOCRUZ, Brazil.

### Ethics statement

The MHOM/BR/75/LTB0016 strain of *L*. *amazonensis* was used throughout this study. This strain was isolated from a human case of cutaneous leishmaniasis in Brazil. This study was performed in strict accordance with the recommendations of the Guide for the Care and Use of Laboratory Animals of the Brazilian National Council of Animal Experimentation (CONCEA). The protocol was approved by the Committee on the Ethics of Animal Experiments of the Instituto Oswaldo Cruz (CEUA-IOC, License Number: L-11/2017). All data were analyzed anonymously.

### Parasites and mice

Promastigotes were cultivated at 26°C in Schneider’s Drosophila medium (pH 6.9) supplemented with 10% fetal calf serum (v/v), 100 μg/mL streptomycin and 100 U/mL penicillin. Parasite maintenance was promoted by passages every 3 days of culture. Female BALB/c mice (8–10 weeks; provided by the Instituto Ciências e Tecnologia em Biomodelos, ICTB/FIOCRUZ) were used in this study. All animals were bred and maintained at the Fundação Oswaldo Cruz according to Guide for the Care and Use of Laboratory Animals of the Brazilian National Council of Animal Experimentation (CONCEA).

### Antimony-resistant induction in *L*. *amazonensis* promastigotes and resistance confirmation

*L*. *amazonensis* promastigotes (MHOM/BR/77/LTB0016) were cultivated following the procedure above with or without addition of potassium antimony tartrate (SbIII) progressively for each passage [27] up to 10 times the previously determined antimony IC_50_ (16 μM). A wild-type control was cultivated in parallel without antimony addition, and both cells reached 32 passages. The resistance was confirmed by incubating antimony-resistant and wild-type promastigotes with increasing concentrations of potassium antimony tartrate (0.3 μM—5000 μM) (S1 Fig). The 50% inhibitory concentration (IC_50_) was determined by logarithmic regression analysis using GraphPad Prism 6 (GraphPad Software, La Jolla, CA, USA). The experiments were performed thrice.

### Promastigote proliferation assay

*L*. *amazonensis* (5x10^6^ /mL) promastigotes (wild type or antimony resistant) were incubated with different concentrations of 2HF (3 μM—96 μM) or vehicle (DMSO 0.2% v/v) for 24 hours. The cell density was estimated using a Neubauer chamber. The growth curve was initiated with 5.0 x 10^6^ cells/ml. The 50% inhibitory concentration (IC_50_) was determined by logarithmic regression analysis using GraphPad Prism 6 (GraphPad Software, La Jolla, CA, USA). The experiments were performed thrice.

### Leishmania-macrophage interaction assay

Peritoneal macrophages were collected from BALB/c mice (8–10 weeks old) and placed into RPMI-1640 medium supplemented with 10% fetal calf serum and plated (2x10^6^ cells/mL) onto Lab-Tek eight-chamber slides with 400 μL in each well for one hour for adhesion (37°C/5% of CO_2_). *L*. *amazonensis* promastigotes (wild type or antimony resistant) were counted, added to the peritoneal macrophages with an MOI (multiplicity of infection) of 5 promastigotes per macrophage, and incubated for 3 hours. The Lab-Tek wells were washed with RPMI-1640 medium after 3 hours of infection to remove non-adherent macrophages as well as promastigotes. After eighteen hours, infected macrophages were incubated with different concentrations of 2HF (0 μM—48 μM) or meglumine antimoniate (0 μM—200 μM) for 72 hours. Lab-Teks were stained with Instat Prov (Newprov, Curitiba/Brazil). The percentage of infected macrophages was determined by light microscopy by counting a minimum of 200 cells. The result was expressed as the infection index (% of infected macrophages × number of amastigotes/total number of macrophages). The IC_50_ value was determined by logarithmic regression analysis using GraphPad Prism 6. In the control samples (absence of 2HF), a similar volume of vehicle (DMSO 0.2% v/v) was added to the cells. The experiments were performed thrice.

### Cytotoxicity assay

Peritoneal macrophages were collected as described above. After 1 hour of adhesion, macrophages were incubated with different concentrations of 2HF (0 μM- 96 μM) without infection for 72 hours (37°C and 5% of CO_2_). The macrophage viability was accessed using resazurin (20% v/v), which was reduced to resorufin after contacting viable cells, and the fluorescence (ex/em: 560/590 nm) was measured by a SpectraMax M2—Molecular Devices, Silicon Valley, USA. The cytotoxicity concentration (CC_50_) was determined by logarithmic regression analysis using GraphPad Prism 6 (GraphPad Software, La Jolla, CA, USA). The experiments were performed thrice.

### 2HF *in silico* evaluation

To predict the pharmacokinetic properties (ADMET—absorption, distribution, metabolism, excretion and toxicity) of 2HF, the ADMETSar tool [28] was used. The SMILES (simplified molecular-input line-entry system) used for *in silico* analysis was as follows: OC1 = CC = CC = C1C1CC (= O)C2 = C(O1)C = CC = C2

### *In vivo* infection in the murine model

To evaluate the *in vivo* effects of 2HF, female BALB/c mice (n = 5 per group, 8–10 weeks old) were infected with wild-type (2x10^6^/10 μL of PBS) or antimony-resistant (4x10^6^/10 μL of PBS) *L*. *amazonensis* promastigotes in the right ear. The treatment started seven days post-infection, with 50 mg/kg/day of 2HF (diluted in DMSO (0.2% v/v), incorporated in an oral suspension) administered orally through an orogastric tube once daily seven times per week until the end of the experiment (day 42), when the animals were euthanized. The control group was treated orally with an oral suspension in DMSO (0.2% v/v) in the absence of 2HF (vehicle of 2HF only). The positive control was treated with intraperitoneal injections of meglumine antimoniate (pentavalent antimonial; 100 mg/kg/day) once daily seven times per week until the end of the experiment (day 42). The lesion sizes were measured twice per week using a dial caliper.

### Parasite load quantification

The parasite load was determined 42 days post-infection using a quantitative limiting dilution assay as described previously [18]. The infected ears were excised, weighed and minced in Schneider's medium with 20% fetal calf serum. The resulting cell suspension was serially diluted. The number of viable parasites in each ear was estimated from the highest dilution that promoted promastigote growth after seven days of incubation at 26°C.

### Toxicology

Before euthanized, BALB/c mice were anesthetized with Ketamin (200 mg/kg) and Xylazine (16 mg/kg) in solution, administered intraperitoneally. Blood was collected (1mL) via cardiac puncture and distributed in EDTA-containing microtubes for hematological analysis or centrifuged for serum obtainment. Both serum (toxicology markers) and total blood (hematological parameters) from the infected BALB/c mice treated as described above were measured by the Program of Technological Development in Tools for Health-PDTIS-FIOCRUZ.

### Statistical analysis

All experiments were performed in three independent triplicates. The data were analyzed using Student’s t-test or analysis of variance (ANOVA), followed by Bonferroni's post-test in GraphPad Prism 6 (GraphPad Software, La Jolla, CA, USA). The results were considered significant when p≤ 0.05. The data are expressed as the mean ± standard error.

## Results

### 2HF effects against *L*. *amazonensis* wild-type and antimony-resistant promastigotes

**Fig 2:**
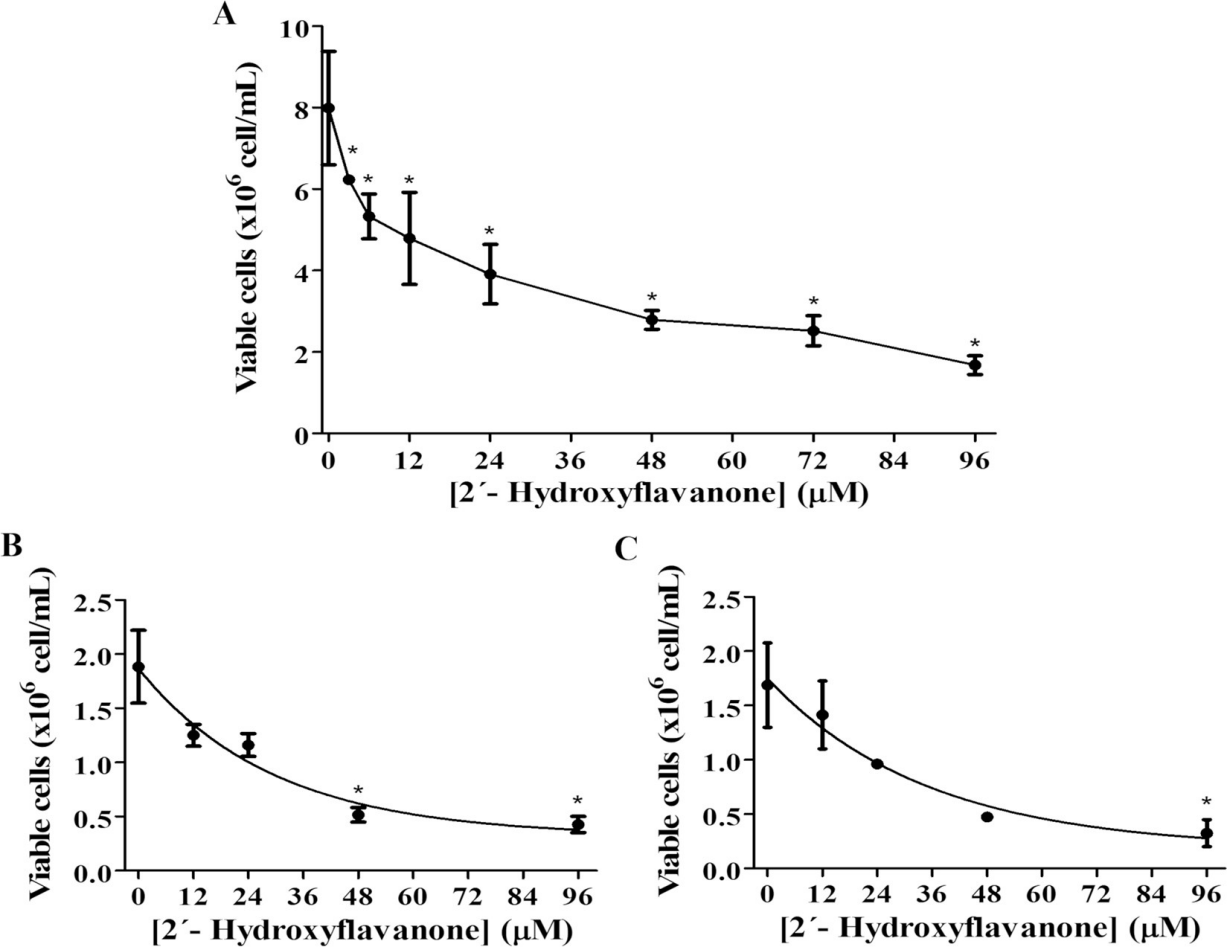
2HF effects against wild-type or antimony-resistant promastigotes. Wild-type or antimony-resistant *L*. *amazonensis* promastigotes were incubated in Schneider’s Drosophila medium in the absence or presence of increasing concentrations of 2HF (3–96 μM) for 24 hours. The number of parasites was determined by direct counting using a Neubauer chamber. In the control (absence of 2HF), the same volume of DMSO (0.2% v/v; solvent of 2HF) was added to the growth medium. The values are presented as the mean ± standard error of three different experiments. a) Wild-type (5 passages), b) Antimony-resistant (32 passages), c) Wild-type comparative (32 passages). The IC_50_ was calculated via nonlinear regression using GraphPad Prism 6.0. * indicates significant difference relative to control (p < 0.05).

2HF demonstrated a dose-dependent inhibition against wild-type *L*. *amazonensis* promastigotes. Over 24 hours of treatment, 2HF was able to inhibit promastigote growth, in addition to killing the parasites in a concentration-dependent manner (0 to 96 μM) with an IC_50_ of 20.96 ± 2.87 μM and achieving 79% inhibition at the highest concentration (96 μM) (Fig 2A).

After antimony-resistant *L*. *amazonensis* promastigotes were obtained (S1 Fig), the effect of 2HF was tested in these cells. Over 24 hours of incubation, the flavanone was able to inhibit the cellular proliferation of the antimony-resistant *L*. *amazonensis* promastigotes (Fig 2B) in a dose-dependent manner similar to that observed with wild-type *L*. *amazonensis* promastigotes, presenting an IC_50_ of 24.34 ± 0.33 μM.

As explained in the Methods section, the antimony-resistant promastigotes were cultivated over several passages, and a wild-type control was cultivated in parallel. To rule out the possibility that the effect observed in the antimony-resistant *L*. *amazonensis* promastigotes was caused by the number of the passages used to induce the resistance, 2HF was also tested against wild-type *L*. *amazonensis* promastigotes with the same number of passages used for the antimony-resistant cells (32 passages). 2HF was capable of inhibiting the cellular proliferation of the wild-type *L*. *amazonensis* promastigotes cultivated with 32 passages with an IC_50_ value of 20.41 ± 0.28 μM, demonstrating no difference in IC_50_ values compared to the IC_50_ values in wild-type *L*. *amazonensis* promastigotes cultivated with 5 passages or antimony-resistant *L*. *amazonensis* promastigotes (32 passages) (Fig 2C). Comparative IC_50_ values are shown in Table 1.

**Table 1:** Comparative IC_50_ for 2HF against wild-type and antimony-resistant *L*. *amazonensis* promastigote.

WT (5 passages)	WT (32 passages)	R (32 passages)
20.96 ± 2.87 μM	20.41 ± 0.28 μM	24.34 ± 0.33 μM

WT: wild-type; R: antimony-resistant

### 2HF is able to reduce wild-type and antimony-resistant *L*. *amazonensis in vitro* infection

Using a peritoneal BALB/c mice macrophage infection model, both pentavalent antimonial (meglumine antimoniate)—a reference drug in leishmaniasis chemotherapy—and 2HF were tested against *L*. *amazonensis*-infected macrophages using wild-type and antimony-resistant *L*. *amazonensis* promastigotes.

**Fig 3:**
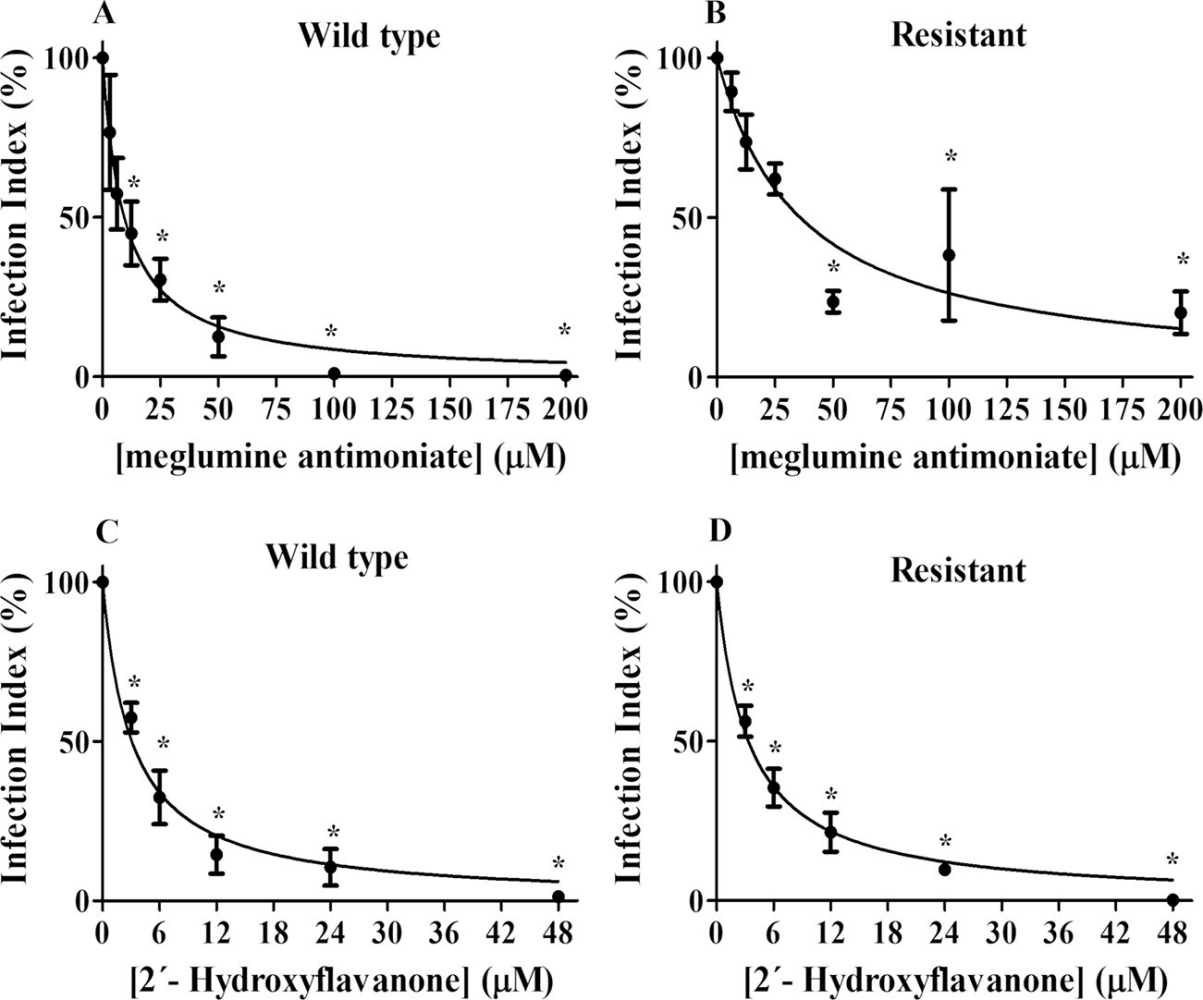
Effect of 2HF and meglumine antimoniate on *L*. *amazonensis*-infected macrophages. Macrophages were infected with wild-type or antimony-resistant *L*. *amazonensis* promastigotes at 37°C and 5% CO_2._ After 3 hours of infection, the remaining promastigotes were removed. After 18 hours, the infected macrophages were incubated in the absence or presence of increasing concentrations of 2HF (3–48 μM) or meglumine antimoniate (3.125–200 μM) for 72 hours. The infection index was determined using light microscopy. At least 200 macrophages were counted on each coverslip in duplicate. The values shown represent the mean ± standard error of three independent experiments. In the control samples (absence of 2HF), a similar volume of vehicle (0.2% DMSO) was added to the cells. Panel A and B: Wild-type and antimony-resistant cells, respectively, treated with meglumine antimoniate; Panel C and D: Wild-type and antimony-resistant, respectively, treated with 2HF. The values are presented as the mean ± standard error of three different experiments. 2HF: 2’-hydroxyflavanone; WT: Wild-type; R: Antimony-resistant; Vehicle: RPMI-1640 medium with 0.2% DMSO. * indicates significant difference relative to control (p < 0.05).

First, to demonstrate that the antimony resistance was not lost in the amastigote transformation process inside the macrophage vacuoles, the effect of meglumine antimoniate was tested. The IC_50_ values for meglumine antimoniate in the wild-type *L*. *amazonensis* and antimony-resistant *L*. *amazonensis* were 9.3 ± 1.38 μM and 35.7 ± 6.57 μM, respectively, demonstrating an almost 4 times resistance (Fig 3A and 3B).

2HF was able to reduce the infection index in both wild-type *Leishmania*-infected macrophages and antimony-resistant *Leishmania*-infected macrophages in a dose-dependent manner (Fig 3C and 3D). The 2HF IC_50_ was 3.09 ± 0.4 μM for wild-type cells and 3.36 ± 0.29 μM for antimony-resistant cells, reaching 99.7% and 99.6% inhibition, respectively, at the highest dose tested (48 μM).

**Fig 4:**
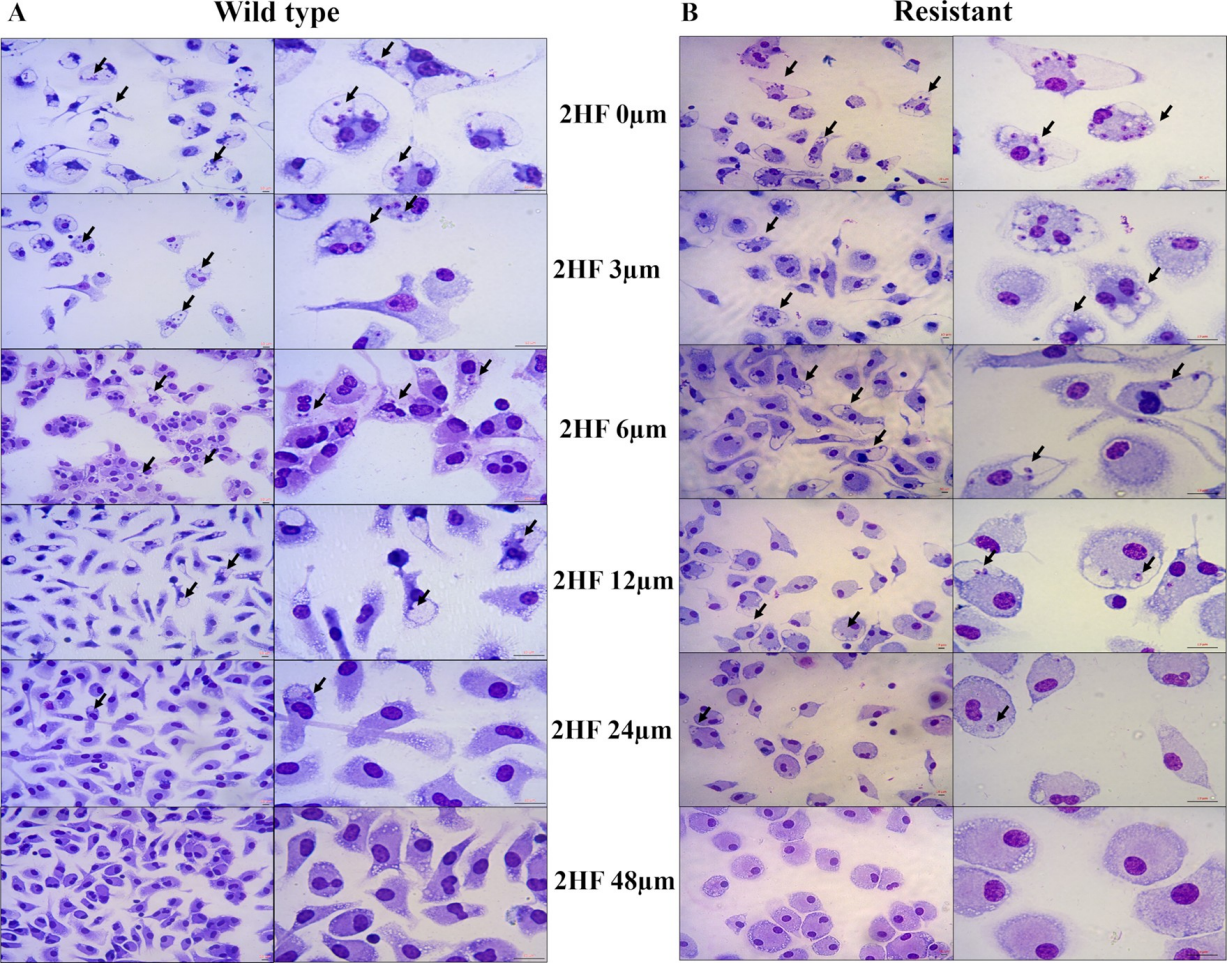
Illustrative photos of 2HF and meglumine antimoniate against *L*. *amazonensis*-infected macrophages. Macrophages infected with wild-type *L*. *amazonensis* promastigotes (Panel A) or antimony-resistant *L*. *amazonensis* promastigotes (Panel B). Scale bars correspond to 10 μm. Black arrows indicate the presence of amastigotes. 2HF: 2’-hydroxyflavanone.

The compound was able to reduce the number of infected cells by over 90% at a concentration of 48 μM. Comparative IC_50_ values are shown in Table 2. The 2HF activity can also be observed in representative photos, showing no macrophage morphology alterations and highlighting the reduced infection index, with almost 100% inhibition at the concentration of 48 μM (Fig 4).

**Table 2:** Comparative IC_50_ of meglumine antimoniate and 2HF against *L*. *amazonensis*-infected macrophages using wild-type and antimony-resistant *L*. *amazonensis* promastigotes.

	Antimonial		2HF	
	WT	R	WT	R
**IC_50_ value**	9.37 ± 1.38 μM	35.73 ± 6.57 μM	3.09 ± 0.40 μM	3.36 ± 0.29 μM

WT: wild-type; R: antimony-resistant

In the evaluation of its possible cytotoxic effects, 2HF demonstrated a CC_50_ of 88.15 ± μM over 72 hours (S2 Fig), and a selectivity index of 28.5 and 26.2 for wild type and antimony-resistant, respectively. The biological efficacy of a drug is not attributed to cytotoxicity when the selectivity index is greater than or equal to 10 [29], indicating that it was not toxic to macrophages at the concentrations used in the infection protocol.

### 2HF demonstrates favorable *in silico* predicted properties

To perform the *in silico* analysis and evaluate the potential of 2HF as a future drug for the treatment of leishmaniasis by the oral route, we used the ADMETSar platform [28] to assess the predicted pharmacokinetic properties (ADMET—absorption, distribution, metabolism, excretion and toxicity) of the compound. We also evaluated its chemical characteristics according to the "Rule of Five" (Ro5) of Lipinski [30,31]. The compound was able to fully satisfy Lipinski’s rule of five, not violating any rule. Upon interpreting the results obtained from the ADMETSar database, 2HF was found to exhibits a high probability of human intestinal absorption, appearing to be permeable to Caco-2 cells and not to be a P-glycoprotein substrate. Regarding metabolism, 2HF is not a CYP substrate but is an inhibitor of CYP2C9, CYP2C19 and CYP1A2. In the toxicity analysis, 2HF demonstrated good probabilities for no Ames toxicity or carcinogenicity (Table 3). Taken together, these data suggest that 2HF is safe and orally absorbed.

**Table 3:** 2HF *in silico* ADMET predictions.

Model	Result	Probability
**Absorption**		
**BBB**	+	90.44%
**HIA**	+	100%
**Caco-2**	+	79.59%
**P-glycoprotein substrate**	NS	57.38%
**P-glycoprotein inhibitor**	NI	81.82%
**Metabolism**		
**CYP450 2C9 Substrate**	NS	73.86%
**CYP450 2D6 Substrate**	NS	88.10%
**CYP450 3A4 Substrate**	NS	64.87%
**CYP450 1A2 Inhibitor**	I	79.45%
**CYP450 2C9 Inhibitor**	I	90.58%
**CYP450 2D6 Inhibitor**	NI	85.61%
**CYP450 2C19 Inhibitor**	I	90.03%
**CYP450 3A4 Inhibitor**	NI	80.55%
**Toxicity**		
**AMES Toxicity**	N	83.11%
**Carcinogens**	N	92.06%
**Lipinski’s rule of 5**		
**HBA (≤10)**	3	**0 violations**
**HBD (≤5)**	1	
**miLogP (≤5)**	3.123	
**n-ROTB (≤10)**	1	
**MW (≤500)**	240.25	

BBB—blood-brain barrier; HIA—human intestinal absorption; + positive;—negative; I—inhibitor; NI—noninhibitor; NS—non-substrate; n-ROTB—number of rotatable bonds; HBA—number of hydrogen bond acceptors; HBD—number of hydrogen bond donors; milogP—logarithm of compound partition coefficient between n-octanol and water; MW—molecular weight.

### 2HF inhibits lesion growth and reduces the parasitic load in experimental wild-type and antimony-resistant cutaneous leishmaniasis

**Fig 5:**
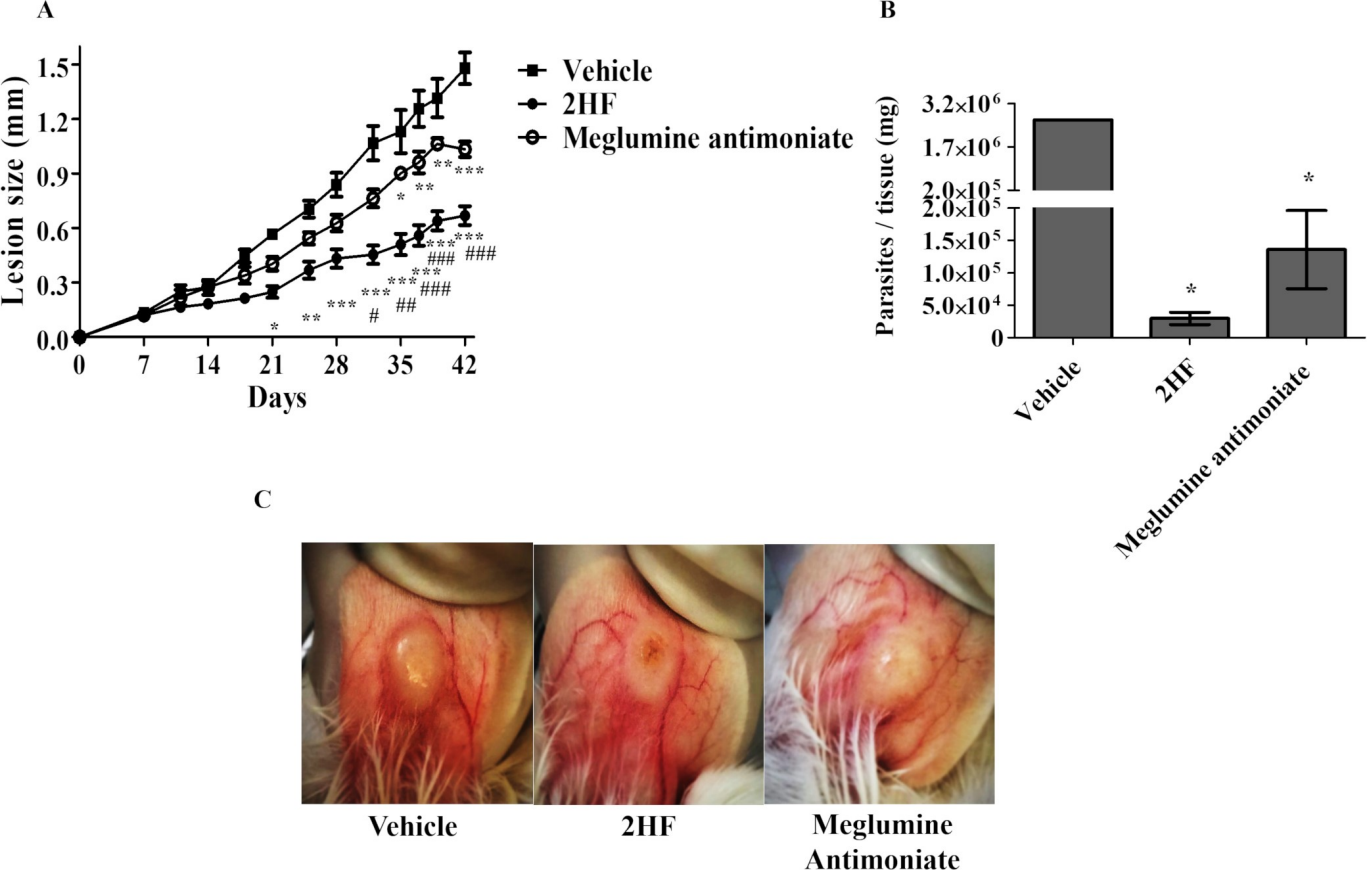
*In vivo* effects of 2HF and meglumine antimoniate using wild-type *L*. *amazonensis*. BALB/c mice were infected in the right ear with 2 × 10^6^ wild-type *L*. *amazonensis* promastigotes. Panel A: Lesion development on the animals treated orally with 2HF (50 mg/kg/day), intraperitoneally with meglumine antimoniate (100 mg/kg/day) and with an oral suspension added to DMSO (0.2% v/v) (2HF vehicle). The treatment started seven days post-infection and was given once daily seven times per week until the end of the experiment (day 42). Panel B: Parasite burden of the *L*. *amazonensis*-infected BALB/c mice untreated or treated with 2HF (50 mg/kg/day) or meglumine antimoniate (100 mg/kg/day). Ear parasite loads were determined via a limiting dilution assay. Data are expressed as the means ± standard errors. These data represent two independent experiments with five mice per group each (n = 5). *, ** and *** indicate significant differences relative to the control group and #, ##, ### indicate significant differences relative to 2HF (p < 0.05; p< 0.01 and p < 0.001, respectively); Panel C: Illustrative lesion photos of a representative infected ear treated with the vehicle (left photo), 2HF (center photo) and meglumine antimoniate (right photo). 2HF = 2’-Hydroxyflavanone.

Taking into consideration the *in vitro* results and favorable *in silico* analysis, 2HF activity was evaluated *in vivo* in a murine model of cutaneous leishmaniasis. In BALB/c mice infected with *L*. *amazonensis* wild-type promastigotes, as shown in Fig 5, the oral administration of 2HF (50 mg/kg/day) reduced the lesion size (p < 0.01) from day 21 post-infection (panel A) and the parasite load (p < 0.001) (panel B), demonstrating 98.8% inhibition by 2HF. Reduction in the lesion size and parasite load, together with illustrative photos of infected ears (Fig 5C), demonstrates the ability of 2HF to control *L*. *amazonensis* infection in BALB/c mice.

Additionally, significant differences between the infected mice treated with 2HF (50 mg/kg/day) and meglumine antimoniate (100 mg/kg/day) were observed in terms of lesion size (p < 0.05) (Fig 5A). However, no statistically significant difference (p = 0.0655) was observed between 2HF (50 mg/kg/day) and meglumine antimoniate (100 mg/kg/day) in terms of parasite load.

Serological toxicology markers such as alanine aminotransferase, aspartate aminotransferase and creatinine were evaluated, and no significant changes were observed, suggesting the absence of liver and kidney toxicity. Additionally, hematological parameters were evaluated and indicated that 2HF did not promote any changes (S1 Table).

**Fig 6:**
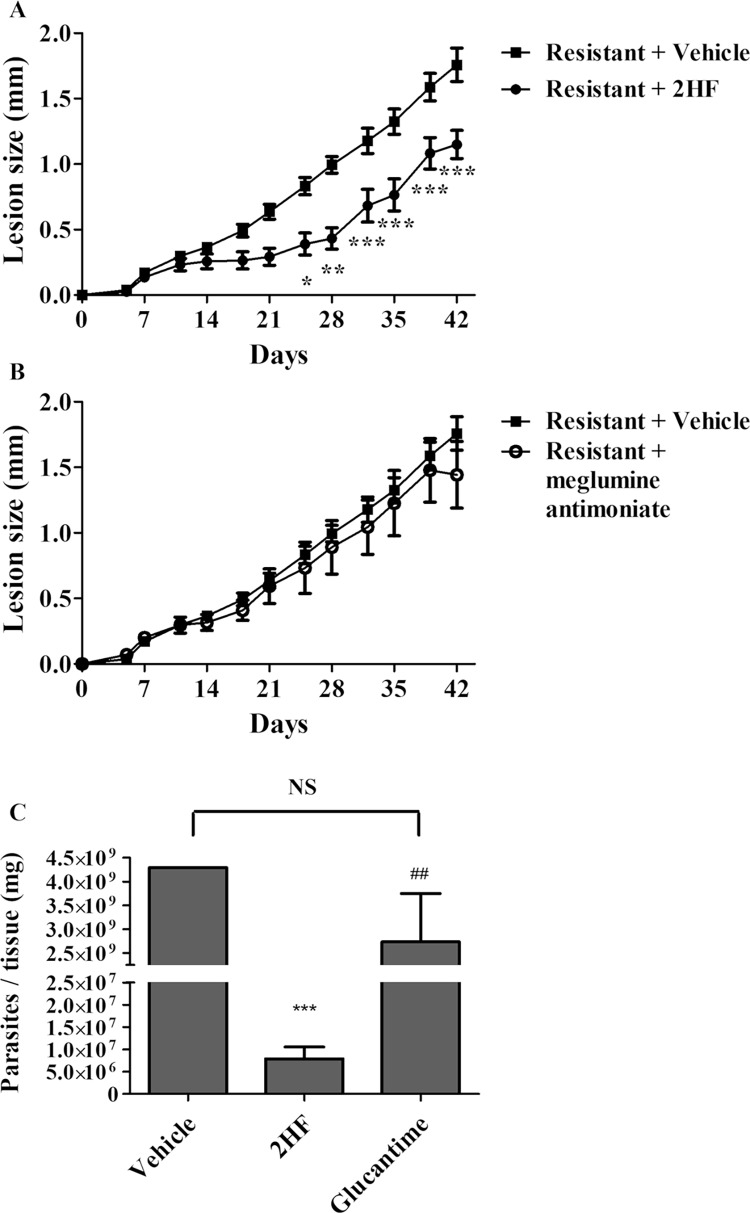
*L*eishmanicidal effect of 2HF and meglumine antimoniate in antimony-resistant *L*. *amazonensis* -infected BALB/c mice. BALB/c mice were infected in the right ear with 4 × 10^6^ antimony-resistant *L*. *amazonensis* promastigotes. Panel A: Lesion development on the animals treated orally with 2HF (50 mg/kg/day). Panel B: Lesion development on the animals treated intraperitoneally with meglumine antimoniate (100 mg/kg/day). The untreated mice (control group) were treated with an oral suspension added to DMSO (0.2% v/v) (2HF vehicle). The treatment started seven days post-infection and was given once daily seven times per week until the end of the experiment (day 42). Panel C: Parasite burden of the *L*. *amazonensis*-infected BALB/c mice untreated or treated with 2HF (50 mg/kg/day) or meglumine antimoniate (100 mg/kg/day). Ear parasite loads were determined via a limiting dilution assay. Data are expressed as the means ± standard errors. These data represent one independent experiment with five mice per group each (n = 5). *, ** and *** indicate significant differences relative to the control group (p < 0.05; p < 0.01 and p < 0.001, respectively) and ## indicate significant differences relative to 2HF (p < 0.01); 2HF = 2’-Hydroxyflavanone; ns = No statistical significance.

Furthermore, 2HF was tested against BALB/c mice infected with antimony-resistant *L*. *amazonensis* promastigotes. 2HF treatment significantly reduced both the lesion size starting from day 25 (p < 0.05) and the parasite load by 99% (p < 0.05) compared the control treatment (Fig 6A and 6C). Moreover, BALB/c mice infected with antimony-resistant promastigotes were also treated with pentavalent antimonial (meglumine antimoniate) (a reference drug used in the treatment of leishmaniasis and a drug used to induce resistance). As observed in Fig 6, meglumine antimoniate was not capable of reducing the lesion size (panel B) and parasite load (panel C), corroborating the parasite resistance.

Hematological and toxicological parameters were analyzed, showing no significant alterations (S2 Table).

**Fig 7:**
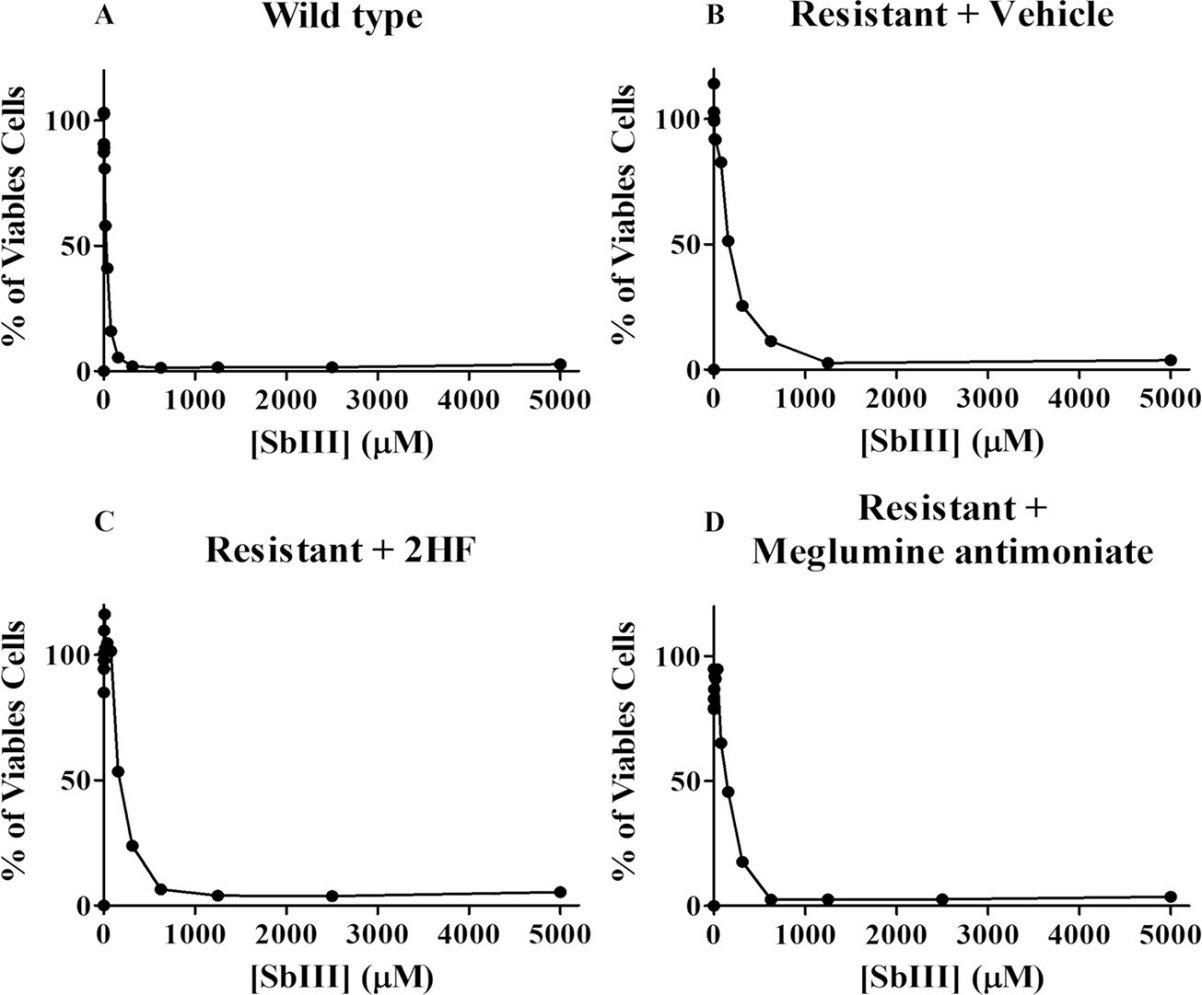
Resistance confirmation in *in vivo* recovered promastigotes. *L*. *amazonensis* promastigotes were recovered from the *in vivo* limiting dilution experiment from each treated group and cultivated with Schneider’s Drosophila medium. Promastigotes were incubated in the presence or absence of the potassium antimony tartrate (SbIII) (0.3–5000 μM) for 72 hours. The viability was measured by resazurin. The IC_50_ for resistance confirmation was calculated via nonlinear regression using GraphPad Prism 6.0. The values are presented as the mean ± standard error of two different experiments. Panel A: Wild-type promastigotes; Panel B: Promastigotes recovered from the vehicle treatment group; Panel C: Promastigotes recovered from the 2HF-treated group; Panel D: Promastigotes recovered from the meglumine antimoniate-treated group.

To confirm the maintenance of resistance, promastigotes were recovered from the infected ears of mice from each treated group (vehicle, 2HF and meglumine antimoniate). These promastigotes were tested for antimony resistance and compared to wild-type *L*. *amazonensis* promastigotes. Wild-type promastigotes presented an IC_50_ of 25.33 μM (Fig 7A). However, promastigotes recovered from the vehicle treatment group demonstrated an IC_50_ of 157.9 ± μM, a 6.2-times antimony resistance compared to wild-type promastigotes (Fig 7B). The 2HF-treated promastigotes demonstrated an IC_50_ of 212 ± μM, an 8.4-times antimony resistance compared to wild-type promastigotes (Fig 7C). Finally, meglumine antimoniate-treated cells showed an IC_50_ of 122 ± μM, a 5-times resistance to antimony compared to wild-type promastigotes (Fig 7D). Taken together, these results confirmed the maintenance of resistance in pentavalent antimony-resistant *L*. *amazonensis* promastigotes after *in vivo* infection. Comparative IC_50_ values are shown in Table 4.

**Table 4:** Comparative IC_50_ for antimonial against *in vivo* recovered promastigotes.

	WT	R + vehicle	R + 2HF	R + A
**IC_50_**	25.33 μM	157.9 μM	212 μM	122 μM
**Resistance fold**	-	6.2	8.4	5

WT: wild-type; R: antimony-resistant; 2HF: 2’-Hydroxyflavanone; A: meglumine antimonial

## Discussion

The current chemotherapy scenario for leishmaniasis suffers from side effects, resistance and high costs [32,33]. Pentavalent antimonial is the first choice for the treatment of leishmaniasis, however antimonial resistance has become a serious problem. Nevertheless, it is still being used in other regions of the world, including Latin America and East Africa [34]. Therefore, the search for new drugs and targets with more efficacies, less toxicity and affordability has recently been increasing.

In an attempt to reduce side effects and resistance, the search for natural products has grown [5] and has highlighted secondary metabolites, especially flavonoids. Flavonoids are polyphenols that are synthesized by plants [14,17]. They have been well known due to their pharmacological properties, including antiviral, anti-inflammatory, antineoplastic, trypanosomicidal and leishmanicidal activities [18–20,22,23,35–38]. Many studies of these metabolites, however, have not advanced beyond *in vitro* assays due to negative results obtained in initial screenings or to *in vitro* toxicity problems. Additionally, many have shown promising results but are still waiting to be tested [5,14].

In accordance with the natural products research trend of drug repurposing, 2HF is a flavanone that has demonstrated promising results against tumor cells. In the present study, we demonstrated that 2HF was effective against *L*. *amazonensis in vitro* and *in vivo* by the oral route, in addition to demonstrating no cross-resistance with antimonials.

2HF demonstrated good activity against the promastigote and intracellular amastigote forms of both wild-type *L*. *amazonensis* (IC_50_ of 20.96 μM and 3.09 μM for promastigotes and intracellular amastigotes, respectively) and antimony-resistant *L*. *amazonensis* (IC_50_ of 24.34 μM and 3.36 μM for promastigotes and intracellular amastigotes, respectively). 2HF was able to cause a decrease in the infection index in a dose-dependent manner, reaching almost 100% for both promastigotes at the highest dose tested (48 μM) without showing toxicity toward the host cell (Fig 2A and Fig 3C).

In previous studies using other flavonoids such as apigenin (flavone), quercetin (flavonol), and epigallocatechin-3-gallate (catechin), similar dose-dependent activities compared to 2HF effects were observed in the promastigote and intracellular amastigote forms of *L*. *amazonensis* and *L*. *braziliensis* [18,19,22,23].

Two hypotheses can be postulated to explained the distinct action of 2HF between promastigotes and intracellular amastigotes: 1) Efficacy of compounds may depend on the developmental stage of the parasite; 2) Macrophages could accumulate higher levels of 2HF. Accordingly, it has been demonstrated that several molecules require lower concentrations to exert a pronounced effect against intracellular amastigotes compared to promastigotes [39–41].

The absence of suitable therapy necessitates the development of novel antileishmanial therapies. In this study, we demonstrated that oral 2HF treatment decreases the lesion size and parasite load *in vivo* using both wild-type *Leishmania* and antimony-resistant *Leishmania*. In addition, 2HF did not alter hematological parameters or serological toxicology markers in the infected mice. However, additional specific toxicity studies, such as genotoxicity, should be done.

It is well known that resistance is a major problem for leishmaniasis chemotherapy, particularly antimony resistance, since antimony is the first line of treatment in several countries. The purpose of this work was to show 2HF not only as a good candidate for leishmaniasis treatment but also as an alternative treatment to address therapeutic failure and resistance. Our data demonstrates that 2HF was able to inhibit antimony-resistant promastigotes (Fig 2) similarly to wild-type cells, in addition to its effect against intracellular amastigotes, reducing the infection index in a dose-dependent manner (Fig 3). The most important result was the observation of its ability to control antimony-resistant *Leishmania* infection in the murine model (Fig 5). This is the first time that the activity of a flavonoid on antimony-resistant *L*. *amazonensis* has been demonstrated.

Considering that 2HF reduced the lesion size and parasite load without compromising the overall health of the infected mice, we suggest this compound as a potential candidate for leishmaniasis chemotherapy for cutaneous leishmaniasis caused by both wild-type and antimony-resistant *Leishmania*. Furthermore, studies should be conducted to determine the ideal dose and therapeutic regimen.

## Supporting information

S1 Fig *L. amazonensis* promastigotes resistance confirmation. Antimony-resistant *L*. *amazonensis* promastigotes were cultivated in the absence or presence of potassium antimony tartrate (SbIII) (0.3–2500 μM) for 72 hours. Cell viability was measured using resazurin. The values are presented as the mean ± standard error of three different experiments. The IC_50_ for resistance confirmation was calculated via nonlinear regression using GraphPad Prism 6.0. The IC50 value was 34.21 μM and 300.5 μM for wild-type and antimony-resistant *L*. *amazonensis* promastigotes, respectively, demonstrating an almost 9 times resistance. The values are presented as the mean ± standard error of two different experiments. Panel A: Wild-type *L*. *amazonensis* promastigotes; Panel B: Antimony-resistant *L*. *amazonensis* promastigotes. * indicates significant difference relative to control (p < 0.05).
(TIF)

S2 Fig Cytotoxicity of 2HF in murine macrophages. Peritoneal BALB/c mice were incubated in the absence or presence of 2HF (0–96 μM) for 72 hours. Cell viability was measured by resazurin. The values are presented as the mean ± standard error of two different experiments. The IC_50_ was calculated via nonlinear regression using GraphPad Prism 6.0. The values are presented as the mean ± standard error of three different experiments. * indicates significant difference relative to control (p < 0.05).
(TIF)

S1 Table Hematological and Biochemical parameters of 2HF effects in wild-type infection model. RBC: red blood cells; MCV: mean corpuscular volume; MCH: mean corpuscular hemoglobin; MCHC: mean corpuscular hemoglobin concentration; ALT: alanine aminotransaminase; AST: aspartate aminotransaminase. The values are presented as the mean ± standard error of two different experiments, five mice per group each (n = 5). Hematological parameters and serological toxicology markers in the infected BALB/c mice treated as described above were measured by the Program of Technological Development in Tools for Health-PDTIS-FIOCRUZ.
(DOCX)

S2 Table Hematological and Biochemical parameters of 2HF effects in antimony-resistant infection model. RBC: red blood cells; MCV: mean corpuscular volume; MCH: mean corpuscular hemoglobin; MCHC: mean corpuscular hemoglobin concentration; ALT: alanine aminotransaminase; AST: aspartate aminotransaminase. The values are presented as the mean ± standard error of one experiment, five mice per group each (n = 5). Hematological parameters and serological toxicology markers in the infected BALB/c mice treated as described above were measured by the Program of Technological Development in Tools for Health-PDTIS-FIOCRUZ.
(DOCX)
