# Supplementary figures and images for: 2'-Hydroxyflavanone activity in vitro and in vivo against wild-type and antimony-resistant Leishmania amazonensis
Source: PLoS Negl Trop Dis. 2018 Dec 6;12(12):e0006930. doi: 10.1371/journal.pntd.0006930 (PMC6283348; doi:10.1371/journal.pntd.0006930)

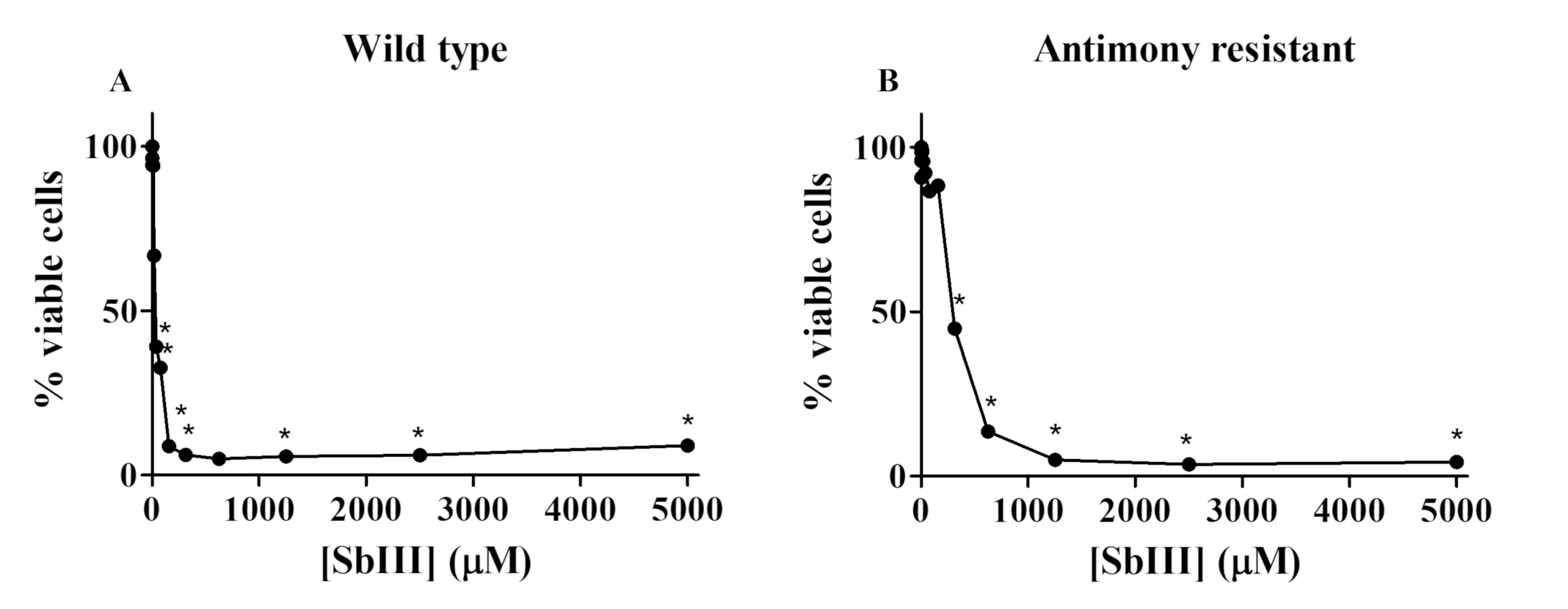

Supplement: S1 Fig — Antimony-resistant L. amazonensis promastigotes were cultivated in the absence or presence of potassium antimony tartrate (SbIII) (0.3–2500 μM) for 72 hours. Cell viability was measured using resazurin. The values are presented as the mean ± standard error of three different experiments. The IC50 for resistance confirmation was calculated via nonlinear regression using GraphPad Prism 6.0. The IC50 value was 34.21 μM and 300.5 μM for wild-type and antimony-resistant L. amazonensis promastigotes, respectively, demonstrating an almost 9 times resistance. The values are presented as the mean ± standard error of two different experiments. Panel A: Wild-type L. amazonensis promastigotes; Panel B: Antimony-resistant L. amazonensis promastigotes. * indicates significant difference relative to control (p < 0.05). (TIF) [file pntd.0006930.s001.tif]

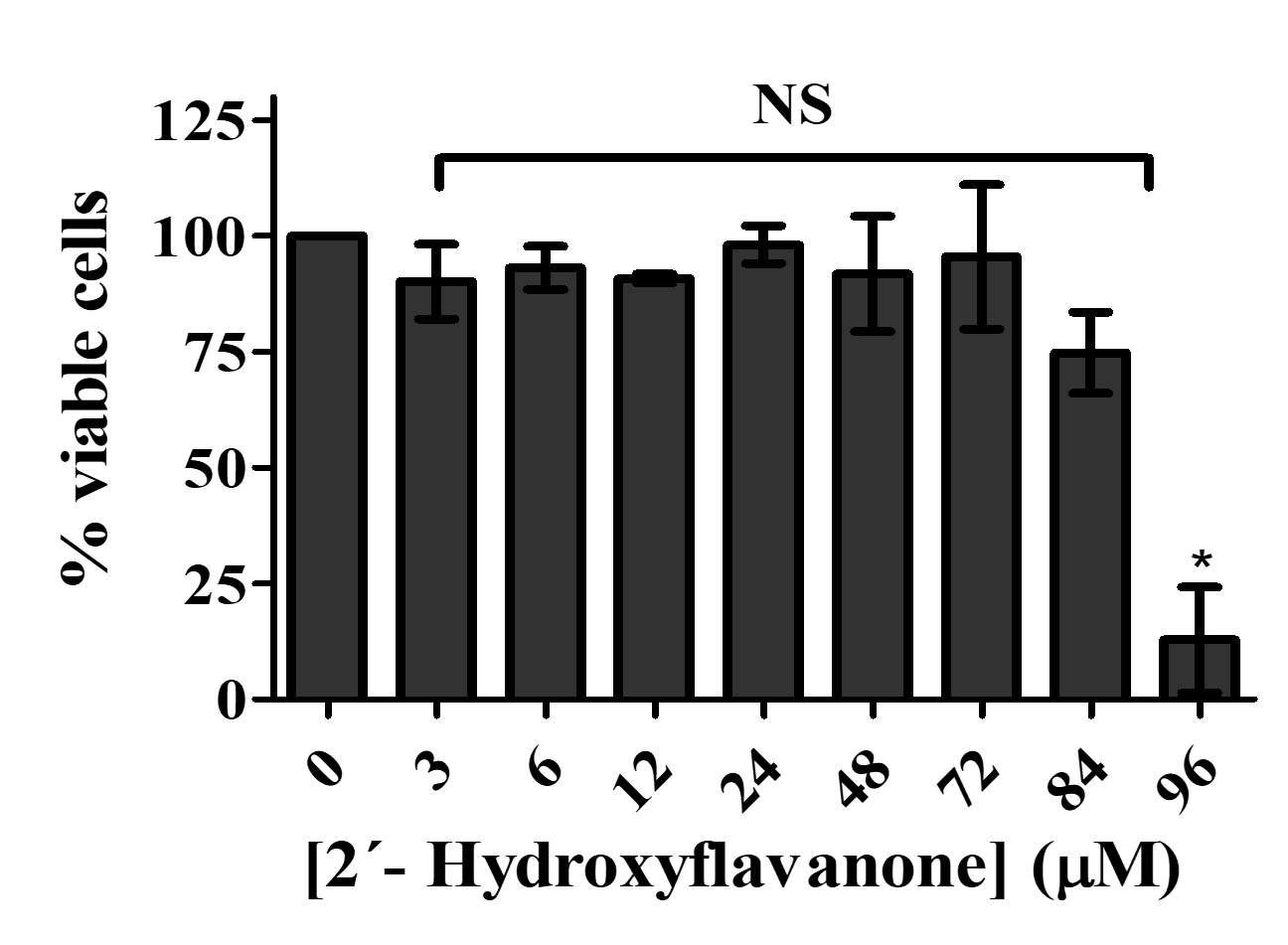

Supplement: S2 Fig — Peritoneal BALB/c mice were incubated in the absence or presence of 2HF (0–96 μM) for 72 hours. Cell viability was measured by resazurin. The values are presented as the mean ± standard error of two different experiments. The IC50 was calculated via nonlinear regression using GraphPad Prism 6.0. The values are presented as the mean ± standard error of three different experiments. * indicates significant difference relative to control (p < 0.05). (TIF) [file pntd.0006930.s002.tif]
