# Supplementary material for: 2'-Hydroxyflavanone activity in vitro and in vivo against wild-type and antimony-resistant Leishmania amazonensis
Source: PLoS Negl Trop Dis. 2018 Dec 6;12(12):e0006930. doi: 10.1371/journal.pntd.0006930 (PMC6283348; doi:10.1371/journal.pntd.0006930)
Supplement: S1 Table — RBC: red blood cells; MCV: mean corpuscular volume; MCH: mean corpuscular hemoglobin; MCHC: mean corpuscular hemoglobin concentration; ALT: alanine aminotransaminase; AST: aspartate aminotransaminase. The values are presented as the mean ± standard error of two different experiments, five mice per group each (n = 5). Hematological parameters and serological toxicology markers in the infected BALB/c mice treated as described above were measured by the Program of Technological Development in Tools for Health-PDTIS-FIOCRUZ. (DOCX) [file pntd.0006930.s003.docx]

**S1 table: Hematological and Biochemical parameters of 2HF effects in wild-type infection model**

|  | Control | 2HF | Meglumine antimoniate |
| --- | --- | --- | --- |
|  |  |  |  |
| RBC (x10^6^ mm^2^) | 11.66 ± 0.094 | 11.35 ± 0.96 | 10.92 ± 0.12 |
| Hemoglobin (g/dL) | 16.32 ± 0.21 | 16.18 ± 0.15 | 15.2 ± 0.15 |
| Hematocrit (%) | 56.34 ± 0.61 | 56.36 ± 0.55 | 54.18 ± 0.94 |
| MCV (fm^3^) | 48.32 ± 0.18 | 50.21 ± 0.28 | 50.27 ± 0.36 |
| MCH (pg) | 14.12 ± 0.14 | 14.37 ± 0.12 | 14.06 ± 0.15 |
| MCHC (g/dL) | 29.0 ± 0.11 | 28.63 ± 0.16 | 28.17 ± 0.30 |
|  |  |  |  |
| Platelets (10^3^/mm^3^) | 1472.3 ± 27.35 | 1356.5 ± 69.78 | 1299.2 ± 30.90 |
| Leucocytes (10^3^/mm^3^) | 6.83 ± 0.22 | 5.88 ± 0.70 | 7.8 ± 1.15 |
|  |  |  |  |
| Creatinine (mg/dL) | 0.10 ± 0.00 | 0.10 ± 0.00 | 0.10 ± 0.00 |
| ALT (U/L) | 54.0 ± 4,73 | 58.25 ± 7.72 | 48.0 ± 3.56 |
| AST (U/L) | 159.3 ±27.09 | 140.0 ± 20.38 | 128.5 ± 7.59 |

RBC: red blood cells; MCV: mean corpuscular volume; MCH: mean corpuscular hemoglobin; MCHC: mean corpuscular hemoglobin concentration ;ALT: alanine aminotransaminase; AST: aspartate aminotransaminase. The values are presented as the mean ± standard error of two different experiments, five mice per group each (n=5). Hematological parameters and serological toxicology markers in the infected BALB/c mice treated as described above were measured by the Program of Technological Development in Tools for Health-PDTIS-FIOCRUZ.
