# Supplementary material for: 2'-Hydroxyflavanone activity in vitro and in vivo against wild-type and antimony-resistant Leishmania amazonensis
Source: PLoS Negl Trop Dis. 2018 Dec 6;12(12):e0006930. doi: 10.1371/journal.pntd.0006930 (PMC6283348; doi:10.1371/journal.pntd.0006930)
Supplement: S2 Table — RBC: red blood cells; MCV: mean corpuscular volume; MCH: mean corpuscular hemoglobin; MCHC: mean corpuscular hemoglobin concentration; ALT: alanine aminotransaminase; AST: aspartate aminotransaminase. The values are presented as the mean ± standard error of one experiment, five mice per group each (n = 5). Hematological parameters and serological toxicology markers in the infected BALB/c mice treated as described above were measured by the Program of Technological Development in Tools for Health-PDTIS-FIOCRUZ. (DOCX) [file pntd.0006930.s004.docx]

**S2 table: Hematological and Biochemical parameters of 2HF effects in antimony-resistant infection model**

|  | Control | 2HF | Meglumine antimoniate |
| --- | --- | --- | --- |
|  |  |  |  |
| RBC (x10^6^ mm^2^) | 9.97 ± 0.005 | 10.06 ± 0.10 | 9.48 ± 0.16 |
| Hemoglobin (g/dL) | 14.35 ± 0.05 | 14.20 ± 0.14 | 14 ± 0.26 |
| Hematocrit (%) | 48.90 ± 0.60 | 48.30 ± 0.57 | 47.60 ± 0.80 |
| MCV (fm^3^) | 49 ± 0.60 | 48.50 ± 0.26 | 49.70 ± 0.20 |
| MCH (pg) | 14.35 ± 0.05 | 14.30 ± 0.06 | 14.60 ± 0.08 |
| MCHC (g/dL) | 29.35 ± 0.25 | 29.40 ± 0.06 | 29.40 ± 0.06 |
|  |  |  |  |
| Platelets (10^3^/mm^3^) | 1192 ± 50 | 1178 ± 57.46 | 1104 ± 77.45 |
| Leucocytes (10^3^/mm^3^) | 8.85 ± 0.35 | 5.90 ± 0.16 | 4.80 ± 0.83 |
|  |  |  |  |
| Creatinine (mg/dL) | 0.10 ± 0.00 | 0.10 ± 0.02 | 0.10 ± 0.02 |
| ALT (U/L) | 47.50 ± 2.12 | 46.50 ± 2.02 | 52 ± 4.49 |
| AST (U/L) | 110.5 ± 17.25 | 78 ± 10.40 | 89 ± 8.15 |

RBC: red blood cells; MCV: mean corpuscular volume; MCH: mean corpuscular hemoglobin; MCHC: mean corpuscular hemoglobin concentration ;ALT: alanine aminotransaminase; AST: aspartate aminotransaminase. The values are presented as the mean ± standard error of two different experiments, five mice per group each (n=5). Hematological parameters and serological toxicology markers in the infected BALB/c mice treated as described above were measured by the Program of Technological Development in Tools for Health-PDTIS-FIOCRUZ.
